# Time after Time: Environmental Influences on the Aging Brain

**DOI:** 10.1289/ehp/122-A238

**Published:** 2014-09-01

**Authors:** Elizabeth Grossman

**Affiliations:** Elizabeth Grossman, a Portland, OR–based environmental and science writer, has written for *Environmental Health News*, *Yale Environment 360*, *Scientific American*, *The Washington Post*, and other publications. Her books include *Chasing Molecules* and *High Tech Trash*.

The population of Americans aged 65 and older is expected to double between 2010 and 2050,^1^ and by midcentury the proportion of the human population made up of people over age 80 is projected to have quadrupled since 2000.^2^ So factors that affect this aging population are of increasing importance. Of particular concern are the neurological diseases and disorders typically associated with advanced age, among them Alzheimer’s and Parkinson’s diseases, dementia, and reduced cognitive function. Investigators are studying the effects of not just present-day exposures and environmental influences such as physical and mental exercise, but also exposures that occurred much earlier in life, whose effects may only become apparent in old age.

**Figure:**
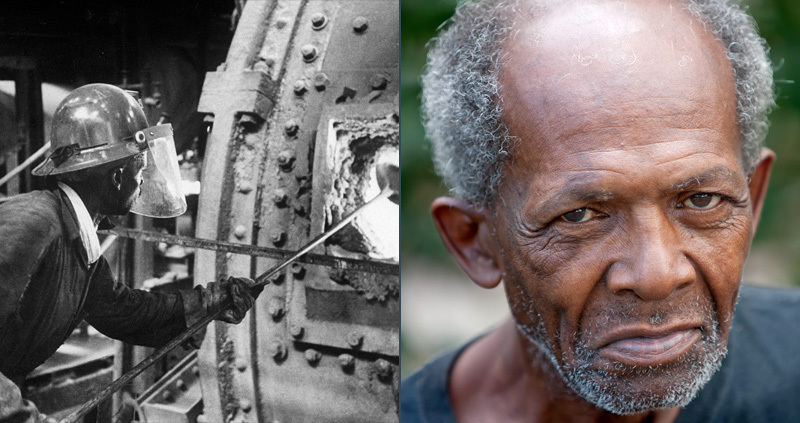
A growing body of evidence suggests that neurological function in the elderly can be affected by exposures from years earlier. Left to right: © Getty; © Corbi

It was long assumed that “once the brain received its allotted quota of nerve cells, its destiny was frozen. After that, the passage of time eroded our allotment steadily and irrevocably,” as professor emeritus Bernard Weiss of the University of Rochester School of Medicine and Dentistry wrote in 2007.^3^ Now, however, there is increasing evidence that the brain is capable of generating new neurons and other functional brain cells even during advanced age. There is also evidence that the older brain can respond quickly and positively to external influences such as physical exercise and intellectual stimulation. This is prompting considerable interest in developing strategies for protecting and enhancing neurological function in the elderly.

The two most vulnerable periods for the brain, Weiss says, are early in life, when the organ is first developing, and later in life, when the body’s defenses and compensatory mechanisms begin to falter. There is a large and growing body of evidence indicating these two vulnerable life stages can be linked when damage incurred during early development contributes to health disorders that may not become apparent until later in life.^4^

Weiss also notes that declining defense mechanisms may magnify vulnerability to contemporary environmental exposures. He says that when older adults experience cognitive problems, diagnoses rarely consider the possibility that environmental chemical exposure may be involved, simply because questions about such exposures are typically not asked as part of clinical intake. Over the past 30 years, Weiss says, research attention has focused primarily on environmental influences on early developmental stages. Far less extensively researched, but a subject of increasing interest, are environmental chemical exposures that can affect the health of the aging brain.

## Neurotoxic Agents

In the past 10 years, however, a number of studies^5^^,^^6^^,^^7^^,^^8^^,^^9^^,^^10^ have looked at the effects of chronic low-level lead exposure on adult humans’ cognitive abilities. The findings of such studies suggest that lead that has accumulated in bones can be mobilized over time as part of the aging process, resulting in exposures that adversely affect adults’ cognitive skills later in life.

One study assessed 466 elderly participants in the VA Normative Aging Study who were environmentally but not occupationally exposed to lead. The study showed that higher bone lead levels were associated with steeper declines in these men’s cognitive skills over several years of followup, even after adjusting for confounding factors.^11^ Other work assessed the impacts of low-level lead exposure combined with self-reported chronic stress in 811 older men participating in the same study. It, too, found these exposures to be associated with impaired cognitive ability.^12^

Other metals may adversely affect neurological function in later life by either acting directly on the brain or adversely impacting other organs or hormones that maintain healthy neurological function. For example, cadmium can cause kidney disease,^13^ which is associated with cognitive problems.^14^^,^^15^ Like lead, cadmium is stored in the body, primarily in the kidneys and liver but also in joints and other tissues, where it has a biological half-time of decades.^16^ Rodent studies indicate cadmium can also interact with the estrogen receptor and interfere with how the body uses calcium and zinc,^17^ both of which play a role in nervous system function.

Similarly, lead and mercury have been associated with liver disease,^18^ which itself is associated with adverse neurological health effects, including a condition that produces a type of neuronal plaque associated with Alzheimer’s disease.^19^ Chemical exposures that adversely affect kidney and liver function can also hamper the body’s ability to detoxify and excrete environmental toxicants, thus letting them remain in the body—an effect that may be particularly problematic in advanced age when a body’s defense mechanisms are in decline.^20^

There is evidence connecting certain metals (e.g., lead, manganese), pesticides (e.g., paraquat, maneb), and solvents (e.g., toluene, trichloroethylene) with neurological symptoms characteristic of Parkinson’s disease. Many of the exposures studied have been occupational, and some were acute, rather than lower-level and chronic. Much more extensive research is needed to determine the precise role environmental exposures to these agents may play in prompting Parkinson’s disease.^21^

**Figure:**
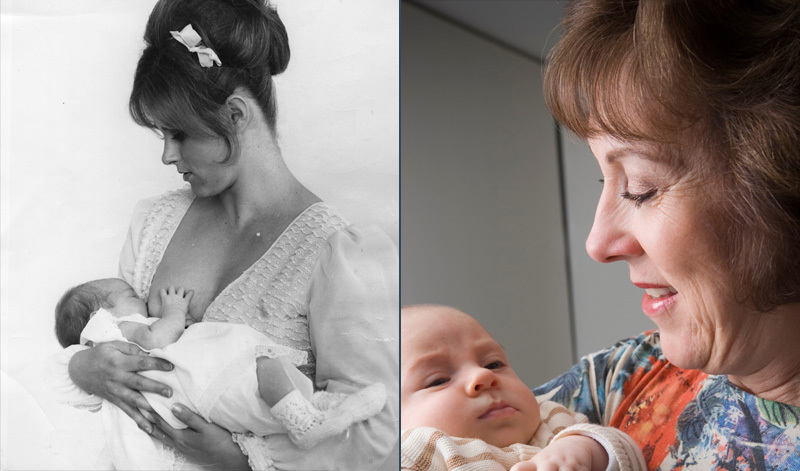
In some cases, contaminants stored in the body are released over time as a function of processes such as pregnancy and aging. In others, damage may occur with no evidence of effects until old age. Left to right: © Brian Eichhorn/Shutterstock; © Getty

More substantial evidence links various solvent exposures to other neurological conditions, including cognitive impairments, neuropathy, and what is sometimes called “pseudodementia,” when temporary neurological dysfunction produces symptoms similar to those of dementia.^22^ Organic solvents, including toluene, have also been found to impair color vision, while other solvent exposures have been linked to hearing loss, particularly when combined with noise exposure.^23^^,^^24^ Such exposures have been primarily studied when they occur occupationally, but some epidemiological studies suggest there is also potential for adverse effects from ambient environmental exposures.^22^

These solvent and pesticide exposures can, of course, occur at any age. But because the neurological disorders with which they are linked mirror those associated with motor and sensory-function declines of aging, they can be mistaken in diagnosis for the effects of aging or diseases of old age like Parkinson’s and Alzheimer’s diseases. ^22^^,^^25^^,^^26^ It also appears that long-term nonacute exposures to solvents and pesticides can affect verbal memory, attention, and spatial skills, with effects that may not become apparent until later in life, when they, too, might be confused with or compounded by aging-related conditions.^22^^,^^27^

More subtle environmental exposures are also thought to be implicated in neurological health effects that can manifest later in life. These include exposures to chemicals that may disrupt the normal function of hormones involved in regulating neurological health, chief among them thyroid hormones.

Hormones are intimately involved with neurological function; a normal brain can’t develop without healthy thyroid hormone function, and the fetal brain is extremely receptive to thyroid hormone.^28^ So if there is early-life thyroid dysfunction, says R. Thomas Zoeller, a professor of biology at the University of Massachusetts Amherst, that person may have cognitive impacts even as an adult.

Thyroid hormones deserve particular attention when considering neurological function in later life because, says Zoeller, these hormones “do different things at different times in the life cycle,” all of which are key to maintaining health. Perturbations in the function of these hormones can produce very subtle subclinical effects—effects that a person would not be aware of in their own body—that nevertheless can set the stage for other health effects much later in life.

Weiss says gonadal hormones (i.e., androgens and estrogens) also deserve far more research attention for their influence on neurological function in the elderly.^29^ These hormones determine sexual differentiation, but they also are involved in neurogenesis and have demonstrated neuroprotective effects in adult male and female animals.^30^

When environmental factors affect thyroid and other hormones, the result can be health effects associated with conditions that impair neurological function. For example, there is evidence that exposure to persistent organic pollutants including dioxins and certain polychlorinated biphenyls, halogenated flame retardants, and pesticides can produce hormonally mediated effects that promote obesity and diabetes, which increase risk for vascular health problems.^31^^,^^32^ There is also evidence that exposures to some of these same compounds may directly increase risk for hypertension and cardiovascular disease.^31^^,^^32^ These cardiovascular conditions can, in turn, cause less dramatic neurovascular effects that sometimes result in memory loss, or what’s called “vascular dementia,” when reduced blood flow to the brain deprives brain cells of oxygen and causes the equivalent of small strokes.^33^

Evidence of similar effects has been reported for exposure to chemicals that are pervasive due to widespread use but are not environmentally persistent. Among these is bisphenol A (BPA). Laura Vandenberg, an assistant professor of environmental health studies at the University of Massachusetts Amherst, explains that numerous animal studies indicate early-life exposure to BPA can produce health effects characteristic of metabolic syndrome.^34^ Individuals with metabolic syndrome are at increased risk for hypertension, with its risk for adverse neurological effects. It is also often hard to exercise for those who are overweight or obese or who have cardiovascular disease or diabetes. Yet aerobic exercise in later life appears to be an essential component of maintaining, if not also enhancing, brain function in older age.^35^^,^^36^^,^^37^

**Figure:**
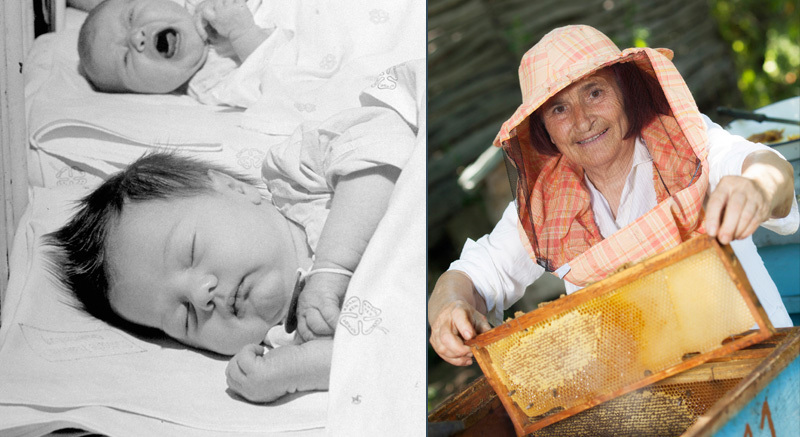
It was long assumed that the brains we were born with were the brains we died with—minus whatever brain cells we lost along the way. There is now evidence that the brain is capable of generating new brain cells even during advanced age. Left to right: © Nina Leen/Getty; © iStockphoto

## Protective Factors

There is now substantial research investigating how physical activity and exercise affect brain function. This is also the area of research where it is perhaps the easiest to make direct comparisons between animal experiments and human studies. As Arthur Kramer, director of the University of Illinois Beckman Institute for Advanced Science and Technology, and colleagues have written, “Abundant data suggests that physical activity reduces the risk of various diseases, including those associated with compromised cognition and brain function (e.g., heart disease, stroke, obesity) and, in turn, independence and quality of life.”^38^

One focus of Kramer’s research is to understand the mechanisms by which exercise protects and restores the brain. He and his colleagues have been studying how physical exercise affects the structure and function of the hippocampus—which plays important roles in memory and in organizing and storing information—and what that means for an individual’s memory capacity. “Anything that’s aerobic seems to have beneficial effects,” Kramer says.

Rodent studies have shown that physical exercise—which is known to increase blood flow to the brain—also appears to increase the generation of new neurons in the hippocampus. This activity furthermore appears to increase synaptic plasticity (which could be described as flexibility and ability to change), angiogenesis (or vascular construction), and levels of neurotrophins (the proteins that regulate nerve cell growth and support neural health).^38^

Of particular interest is learning how physical exercise increases the production of new neurons, and how that may enhance performance of certain memory functions. Functions of interest include what’s called “relational binding”—for example, remembering the name of a person you recently met and where you met that person. Physical exercise also appears to enhance “visual pattern separation,” which enables you to distinguish and remember different patterns—a process that increases memory accuracy. Both functions involve the dentate gyrus region of the hippocampus, which is especially susceptible to age-related changes.^39^

Some studies have reported a doubled or even tripled ability of the dentate gyrus to generate new neurons in rodents that exercised.^40^^,^^41^ Growth of new dendritic spines, which are important for learning and memory, appears to be stimulated as physical or aerobic exercise increases the expression of genes associated with regulating the secretion of neurotrophin proteins, particularly brain-derived neurotrophic factor, says Kirk Erickson, principal investigator of the University of Pittsburgh Brain Aging and Cognitive Health Laboratory. One hypothesis for this, he explains, is that because exercise stimulates blood flow, it may also increase available levels of brain-derived neurotrophic factor.

In experiments with mice, aerobic exercise has been associated with improved spatial memory.^38^^,^^40^ Such activity has also been associated with increased hippocampus size, Erickson explained in a talk at the 2014 annual meeting of the American Association for the Advancement of Science,^42^ and “no pharmaceutical treatment has been able to replicate this effect.” According to Kramer and Erickson, findings from human studies that examined the effects of brisk walking and other aerobic activity have been consistent with those in animal studies.^43^

**Figure:**
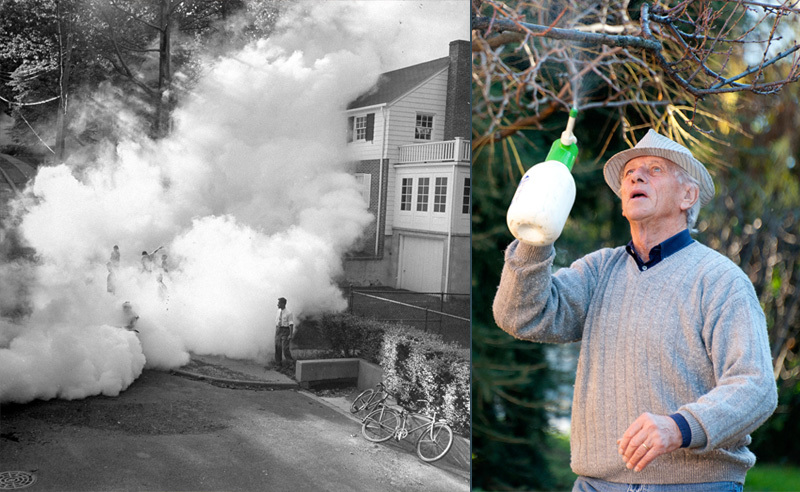
Although both past and present-day exposures can affect neurological function, staying active physically and mentally could play an important role in preserving and even boosting brain function. Left to right: © George Silk/Getty; © Rudi Gobbo/Getty

Physical exercise may also contribute to increased angiogenesis, and increased blood flow to the hippocampus, in turn, is associated with improved cognitive function.^38^^,^^44^^,^^45^^,^^46^ A study that used magnetic resonance imaging to examine cerebral blood vessels found that highly active elderly adults (those who had engaged in aerobic activity for at least 180 minutes a week for the past 10 consecutive years) had brain blood vessel structures similar to those of younger people.^47^ The authors pointed out that it was unclear from this study whether aerobic activity had caused the anatomical difference or whether individuals with “younger” brains had been more likely to be physically active.

“Overall,” wrote Kramer and colleagues in a 2013 review of the evidence on exercise and brain plasticity, “converging evidence suggests exercise benefits brain function and cognition across the mammalian lifespan, which may translate into reduced risk for Alzheimer’s disease in humans.”^38^ A phase I/II clinical trial of Parkinson’s disease patients by these authors suggested that exercising aerobically even for 45 minutes three times a week may markedly improve brain function.^48^

Another important component of maintaining optimal brain function into old age is what is known as cognitive reserve, the brain’s ability to optimize performance and compensate for any brain damage.^49^ Research by Yaakov Stern, director of the Cognitive Neuroscience Division at the Columbia University College of Physicians and Surgeons, suggests that “exercise changes the brain itself,” Stern says, potentially increasing the size of important brain areas that are responsible for synaptic plasticity and enhancing neurovascular function. But in addition to physical exercise, this research indicates that intellectual and social stimulation can potentially increase brain reserve, or the physical structure of the organ.^50^^,^^51^ However, the mechanisms by which this happens are not yet understood well enough to design interventions, in part because it’s difficult to extrapolate animal findings in this area to human experience.

In studies with human adults, Stern and colleagues are measuring what is called efficiency and capacity, or how hard an individual must work to accomplish a particular cognitive task. They are using magnetic resonance imaging to determine what is happening physically in the brain as individuals think their way through the task.^50^^,^^51^ These researchers are also examining what happens to the brain when it activates compensatory neural networks to make up for the lack of function in others. Part of this research involves trying to understand why some people have better efficiency and capacity and more effective compensatory networks, and also why some proceed into later life with more robust cognitive reserves.

One question being explored is whether cognitive flexibility (the ability to structure information in different ways that is key to analytic thinking) and brain plasticity are enhanced by the stimulation that comes with formal education. Another is how brain flexibility later in life is influenced by cognitive stimuli and other factors over a lifetime and at particular life stages. In a new study, Stern and colleagues plan to look at the combined effects of physical and cognitive stimulation to see if they have additive or synergistic effects. It is clear the older brain responds positively to cognitive or intellectual stimulation, but it is not yet clear, explains Stern, whether or how particular games, puzzles, or other memory tasks actually build cognitive reserve. There is evidence, however, to suggest that older people who are active socially and intellectually do enjoy better cognitive function.^52^^,^^53^^,^^54^

At this point, there may be more research questions than answers, but evidence thus far strongly suggests environmental factors can play an instrumental role in influencing neurological function in older adults. Chemical exposures can produce health effects that set the stage for neurological disease and disorders, while physical and intellectual exercise foster brain flexibility and a healthy cognitive reserve. And although investigators have not yet pinpointed how interventions should be designed to produce maximum benefits, so convinced is Kramer of the positive effects of physical and aerobic exercise on neurological health that he believes exercise can reverse, at least temporarily, some of the negative effects of aging on cognitive and brain health.
